# Comparative Evaluation of Two Doses of IV Dexamethasone for Postoperative Analgesia in Patients Undergoing Lower Segment Cesarean Section Under Spinal Anesthesia: A Randomized Controlled Trial

**DOI:** 10.7759/cureus.75020

**Published:** 2024-12-03

**Authors:** Nikitha Vasan, Meenakshi Kumar, Sushil Guria, Krishika Verma, Renuka Choudhary

**Affiliations:** 1 Anesthesiology, Vardhman Mahavir Medical College and Safdarjung Hospital, Delhi, IND; 2 Anesthesiology and Critical Care, Vardhman Mahavir Medical College and Safdarjung Hospital, Delhi, IND; 3 Anesthesiology, Critical Care and Pain Medicine, Vardhman Mahavir Medical College and Safdarjung Hospital, Delhi, IND

**Keywords:** analgesia, cesarean section, dexamethasone, obstetric anesthesia, post-operative pain, spinal anesthesia

## Abstract

Introduction

Effective postoperative analgesia following lower segment cesarean section (LSCS) is crucial for promoting surgical recovery and fostering maternal-neonatal bonding. This study aimed to compare the efficacy of two IV dexamethasone doses (8 mg and 4 mg) in managing postoperative pain in LSCS patients. The objective was to assess whether the 4 mg dose provides comparable pain relief to the 8 mg dose, with the goal of identifying the optimal dosage for effective pain management with minimal side effects.

Methods

This prospective, randomized, interventional comparative study was conducted on 70 parturients undergoing LSCS under spinal anesthesia (SA). The participants were randomly assigned into two groups of 35 each. Group A received 8 mg of IV dexamethasone, while Group B received 4 mg intravenously after the delivery of the baby. The Visual Analogue Scale (VAS) score, time to first rescue analgesia, total rescue analgesic consumption within 24 hours, duration of sensory and motor blockade, incidence of postoperative nausea and vomiting (PONV), and blood sugar levels were measured every six hours up to 24 hours post-surgery for both groups.

Results

The results revealed a significantly lower VAS score in Group A compared to Group B (p < 0.05). However, the incidence of PONV and the duration of sensory and motor blockade were similar between the two groups (p > 0.05). Blood sugar levels were higher in Group A at all time points (p < 0.05).

Conclusions

The 4 mg dose of dexamethasone appears to be a better alternative for postoperative analgesia compared to the 8 mg dose in patients undergoing LSCS under SA. It was associated with a lower mean VAS score, a reduced incidence of PONV, and a smaller increase in blood sugar levels.

## Introduction

Managing postoperative pain is a fundamental aspect of anesthesia practice and an essential humanitarian need following surgical interventions. Effective pain control not only alleviates suffering but also shortens hospital stays, promotes earlier mobilization, and enhances patient satisfaction [1]. The primary goal of postoperative pain management is to reduce pain while minimizing side effects. This can be achieved through multimodal analgesia, incorporating regional anesthetic techniques, NSAIDs, β-blockers, and glucocorticoids [2].

Dexamethasone, a potent and long-acting glucocorticoid, is thought to have analgesic properties. It works by modulating inflammation at the wound site, primarily through prostaglandins and bradykinin, which help prevent a decrease in pain threshold, reduce tissue edema, and mitigate nerve compression caused by inflamed tissues [3,4].

Severe postoperative pain after a cesarean section can disrupt sleep, cause frequent nighttime awakenings, impair daytime functioning, and hinder activities such as breastfeeding. These effects can lead to psychological distress, poor infant care, and impaired maternal-infant interactions [5]. While some studies have highlighted the role of dexamethasone in relieving postoperative pain after lower segment cesarean section (LSCS) under spinal anesthesia (SA), there is limited literature on the exact doses required for optimal effect [6]. Some studies suggest that an 8 mg dose of dexamethasone is sufficient to prolong postoperative analgesia after LSCS, but it may be associated with side effects such as elevated blood sugar and delayed wound healing [7]. Therefore, we conducted this study to compare 8 mg of IV dexamethasone with 4 mg to determine the optimal dose for postoperative analgesia after LSCS under SA.

## Materials and methods

After receiving approval from the Institutional Ethics Committee (registration number CTRI/2021/09/036984, Institutional Ethics Committee: IEC/VMMC/SJH/Thesis/2020-11/CC-34), we conducted a prospective, randomized, interventional comparative study involving 70 parturients. The study was conducted from October 1, 2021, to August 31, 2022, at Vardhman Mahavir Medical College and Safdarjung Hospital, Delhi, India. Pregnant women aged 18-40 years, classified as American Society of Anesthesiologists (ASA) physical status II, and undergoing LSCS were included in the study. Exclusion criteria included patients on glucocorticoid and/or opioid therapy, those with a history of drug allergy to dexamethasone or tramadol, known cases of gestational diabetes, pregnancy-induced hypertension, thyroid disorders, or other high-risk pregnancies.

The primary aim of this study was to evaluate the analgesic efficacy of two doses of IV dexamethasone for postoperative pain relief in patients undergoing LSCS, measured using the Visual Analogue Scale (VAS) score. Secondary objectives included assessing the time to first rescue analgesia, total dose of rescue analgesia, duration of sensory and motor blockade following SA, the effect of different dexamethasone doses on blood sugar levels, and the incidence of postoperative nausea and vomiting (PONV).

**Figure 1:**
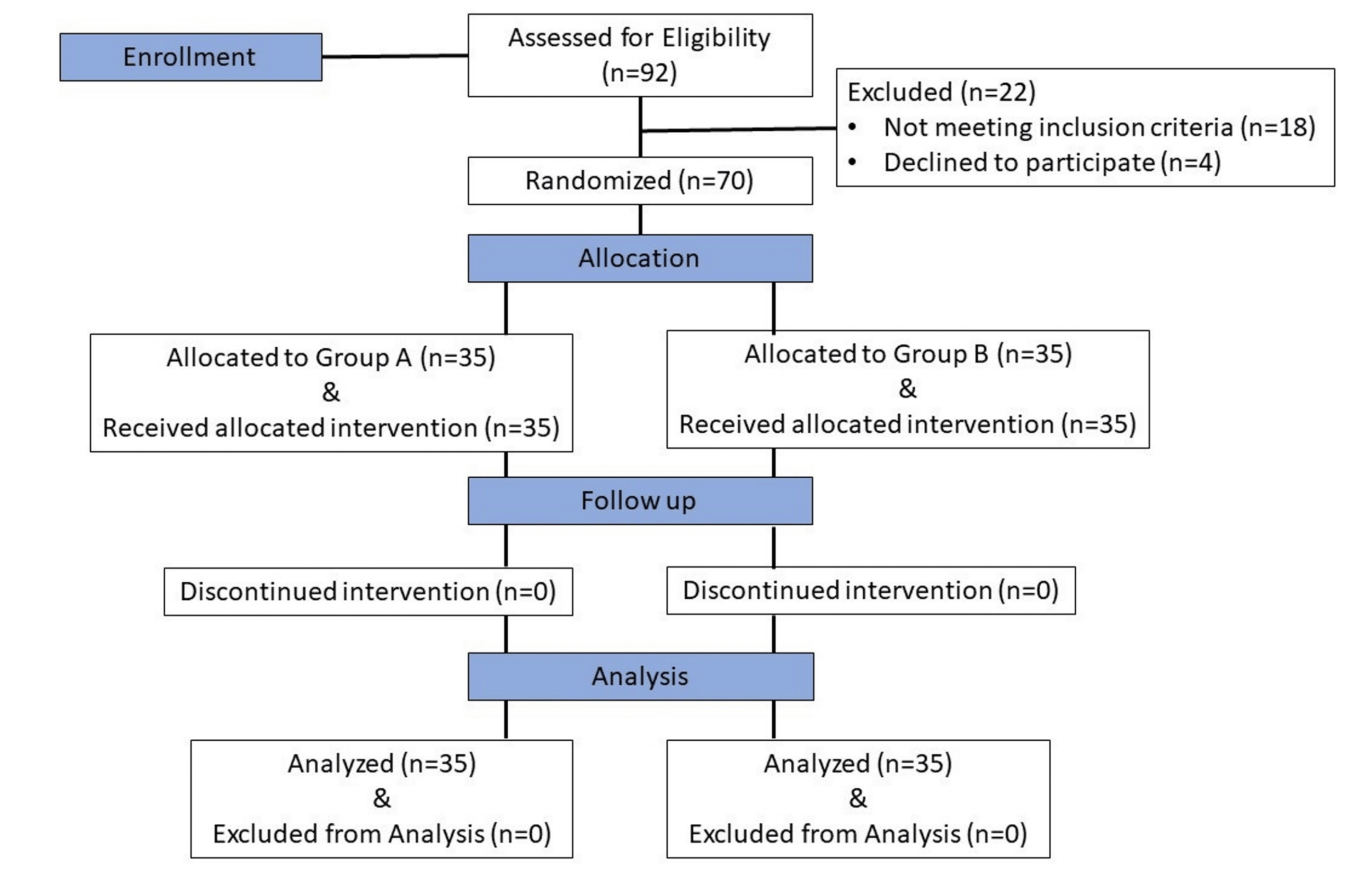
CONSORT flow diagram of patients who received IV dexamethasone after SA SA, spinal anesthesia

Based on previous studies and using the VAS score as the primary endpoint, we calculated a sample size of 70 patients to achieve 90% power with a 5% α-error [8]. Patients were randomized using block randomization with blocks of five. Group A (n = 35) received 8 mg IV dexamethasone, while Group B (n = 35) received 4 mg IV dexamethasone. The CONSORT flow diagram (Figure 1) shows that 92 patients were assessed for eligibility, 70 of whom were enrolled in the study and randomized into two groups of 35 each.

All patients who met the inclusion criteria underwent pre-anesthetic evaluation, and written informed consent was obtained. Patients were also familiarized with the VAS for pain assessment. Subarachnoid block was performed using 2-2.2 ml of injectable bupivacaine 0.5% (hyperbaric) with a Quincke needle (25G) under sterile precautions, after confirming free flow of CSF, to achieve a sensory block up to the T4 dermatome. After delivery, patients in Group A received 8 mg, and patients in Group B received 4 mg of IV dexamethasone.

Post-surgery, patients were transferred to the post-anesthesia care unit for monitoring. Pain severity was assessed using VAS scores at 0, 1, 2, 4, 6, 12, 18, and 24 hours after surgery. A VAS score of 1-3 was classified as mild pain, 4-6 as moderate pain, and 7-10 as severe pain. IV tramadol 50 mg was administered for a VAS score greater than 4. If pain did not subside within 20 minutes, an additional 25 mg of tramadol was given. The time to first rescue analgesia and the total tramadol dose consumed within 24 hours were recorded for each group.

Sensory blockade was assessed using the pin-prick test, and motor blockade was evaluated using the modified Bromage scale at 30-minute intervals until complete recovery from SA. Blood sugar levels were measured at the end of surgery and every six hours thereafter for 24 hours. Any adverse events, including PONV or allergic reactions, were noted and treated according to hospital protocols.

Statistical analysis was performed using IBM SPSS Statistics for Windows, Version 25.0 (Released 2017; IBM Corp., Armonk, NY, USA). The normality of the data was assessed using the Shapiro-Wilk test, which indicated that the data were normally distributed. Quantitative variables were summarized as mean ± SD and compared using the Student’s unpaired t-test, while qualitative variables were summarized as percentages and compared using the chi-square test or Fisher’s exact test, as appropriate. Results were considered statistically significant if the p-value was less than 0.05.

## Results

The demographic profiles of the patients were comparable between both groups (Table 1).

**Table 1:** Demographic parameters of the patients

Variables	Group A (n = 35)	Group B (n = 35)	p-value
Age in years (mean ± SD)	24.89 ± 3.26	25.17 ± 3.01	0.696
Primigravida (%)	20 (57.1%)	18 (51.4%)	0.631
Multigravida (%)	15 (42.9%)	17 (48.6%)	
Weight in kg (mean ± SD)	59.83 ± 5.75	59.60 ± 5.00	0.887
Height in cm (mean ± SD)	153.34 ± 5.46	153.20 ± 4.99	0.967
BMI (kg/m^2^) (mean ± SD)	25.27 ± 1.57	25.36 ± 0.83	0.510

There were no statistically significant differences in perioperative heart rate, mean arterial pressure, respiratory rate, and SpO2 values between the two groups. The mean duration of surgery (p = 0.520), as well as the duration of sensory block (p = 0.233) and motor block (p = 0.220), were also comparable between the groups (Table 2). The time to first rescue analgesia was 5.49 ± 1.11 hours in Group A and 5.31 ± 1.19 hours in Group B, with no statistically significant difference (p = 0.51). The total dose of tramadol administered as rescue analgesia was also similar between the groups, with 96.43 ± 29.79 mg in Group A and 100.0 ± 11.25 mg in Group B (p = 0.50) (Table 2).

**Table 2:** Duration of sensory and motor block, time to first rescue analgesia (tramadol), and total dose of tramadol administered as rescue analgesia in both groups

Variables	Group A (n = 35)	Group B (n = 35)	p-value
	(Mean ± SD)	(Mean ± SD)	
Duration of surgery (minutes)	48.14 ± 4.86	48.86 ± 5.16	0.520
Duration of sensory block (minutes)	144.80 ± 7.51	143.00 ± 9.17	0.233
Duration of motor block (minutes)	162.86 ± 11.00	159.86 ± 10.11	0.220
Time to first dose of tramadol (hours)	5.49 ± 1.11	5.31± 1.19	0.51
Total dose of tramadol (mg)	96.43 ± 29.79	100 ± 11.25	0.5

There was a statistically significant difference in VAS scores between the two groups at the second hour (p = 0.022), fourth hour (p = 0.034), sixth hour (p = 0.048), and 12th hour (p = 0.040). The mean VAS scores were slightly lower in Group A at the second, fourth, and 12th hours compared to Group B, although both groups remained in the same category with VAS scores <4. At the 18th and 24th hours, the values were comparable between the groups (Table 3).

**Table 3:** Comparison of VAS scores between the two groups ^*^ Significant difference in mean between Group A and Group B VAS, Visual Analogue Scale

VAS	Group A	Group B	p-value
	Mean ± SEM	Mean ± SEM	
0 hour	0.00 ± 0.00	0.00 ± 0.00	-
1 hour	1.23 ± 0.08	1.40 ± 0.10	0.183
2 hours	1.89 ± 0.14	2.49 ± 0.19	0.022^*^
4 hours	3.23 ± 0.19	3.8 ± 0.17	0.034^*^
6 hours	3.68 ± 0.30	2.71 ± 0.37	0.048^*^
12 hours	2.31 ± 0.21	2.83 ± 0.20	0.040^*^
18 hours	2.48 ± 0.14	2.77 ± 0.18	0.211
24 hours	2.46 ± 0.09	2.71 ± 0.12	0.133

Two patients (5.7%) in Group A and three patients (8.6%) in Group B experienced PONV immediately after surgery. Additionally, two patients (5.7%) in each group had PONV during the first hour post-surgery. No further incidence of PONV was observed in either group thereafter. There were no differences in blood sugar levels between the two groups at baseline and 0 hours. However, blood sugar levels were significantly higher in Group A compared to Group B at six, 12, 18, and 24 hours (p < 0.001), with only minor differences observed at the 18- and 24-hour intervals (Table 4).

**Table 4:** Comparison of blood sugar values between the two groups ^*^ Significant difference in mean between Group A and Group B BS, blood sugar

BS (mg/dl)	Group A	Group B	p-value
	Mean ± SD	Mean ± SD	
Baseline	99.29 ± 7.30	100.00 ± 7.67	0.691
0 hours	108.11 ± 6.34	107.54 ± 7.62	0.734
6 hours	137.23 ± 7.47	124.80 ± 7.68	<0.001^*^
12 hours	130.29 ± 6.25	121.34 ± 6.38	<0.001^*^
18 hours	123.09 ± 5.64	116.57 ± 6.02	<0.001^*^
24 hours	117.89 ± 5.18	110.46 ± 6.55	<0.001^*^

## Discussion

Adequate postoperative analgesia is essential for the surgical recovery of obstetric patients, facilitating breastfeeding, early ambulation, and better maternal-neonatal bonding. Multimodal analgesia ensures optimal pain management with reduced opioid dependence by employing drugs that act through different mechanisms. Dexamethasone, a long-acting glucocorticoid, not only provides postoperative analgesia through its potent anti-inflammatory effects but also prevents PONV. However, it can elevate blood glucose levels, which may impair wound healing and increase the risk of surgical site infections. Our study aimed to determine an optimal dose of IV dexamethasone for pain relief that would be both effective and safer, with minimal side effects.

The demographic profile and hemodynamic parameters, all within normal limits, showed no significant differences between the groups. While the mean VAS scores in Group A were consistently lower than those in Group B across all time points (except at six hours), statistical significance was observed at two, four, and 12 hours (p < 0.05). Both 8 mg and 4 mg doses maintained VAS scores below 4 postoperatively for 24 hours, indicating mild pain and clinical comparability. The higher mean VAS score in Group A at six hours and the lower score in Group B (p < 0.05) may be attributed to the earlier administration of rescue analgesia in Group B (at 5.31 hours after SA) compared to Group A (at 5.49 hours).

The 8 mg dose of dexamethasone has been extensively studied for its effectiveness in postoperative analgesia, with several authors noting lower VAS scores in the dexamethasone group compared to saline or placebo in patients undergoing LSCS under SA [7-9]. Melese et al. concluded that preoperative administration of dexamethasone 0.1 mg/kg intravenously reduces postoperative pain in pregnant women undergoing LSCS (p = 0.015) [10]. In a comparative study between doses of 8-10 mg and 4 mg of IV dexamethasone, Rahimzadeh et al. found that the higher dose group had a significantly lower mean VAS score compared to the 4 mg group (p < 0.001) [11]. However, in a meta-analysis by Waldron et al., the researchers concluded that dexamethasone provided a statistically significant analgesic benefit compared to placebo, and subgroup analysis of 24-hour pain scores showed no difference between doses of 4-5 mg and 8-10 mg of IV dexamethasone (p = 0.120), which is consistent with the clinical comparability observed in the current study [12].

Similarly, the time to the first dose of rescue analgesia in Group A (5.49 hours) and Group B (5.3 hours) demonstrated similar time frames in clinical practice, with no statistically significant difference (p = 0.51). Several authors have reported that the mean time to request the first dose of rescue analgesia was six to eight hours in patients who received 8 mg IV dexamethasone immediately after SA and three to four hours in the placebo group (p < 0.05) [7,8]. The disparity in our study may be attributed to differences in the timing of dexamethasone administration. Glucocorticoids typically exert their effect by altering protein synthesis via gene transcription, leading to an onset of action within one to two hours [13]. In this study, dexamethasone was administered immediately after delivery, which likely delayed its peak analgesic effect.

Several studies have reported that dexamethasone enhances postoperative analgesia after SA by reducing 24-hour morphine consumption and prolonging the time to the first request for analgesia [6,9,14]. At the end of 24 hours post-surgery, the total rescue analgesic (tramadol) consumption in Group B, receiving 4 mg of dexamethasone, was slightly higher at 100.00 ± 22.76 mg compared to 96.43 ± 29.79 mg in Group A (p = 0.5). This difference was not statistically significant.

There were no clinical or statistically significant differences between the two doses of dexamethasone regarding the duration of sensory (p = 0.233) or motor block (p = 0.220). Shalu and Ghodki reported an increase in the duration of the sensory block with 8 mg IV dexamethasone compared to saline [7], but Parthasarathy et al. found no significant difference in the sensory block duration between the 8 mg and saline groups [8]. Neither of these studies demonstrated a difference in motor blockade after SA [7,8]. Therefore, it is clear that IV dexamethasone does not prolong motor blockade, which could otherwise hinder patient recovery.

The incidence of PONV was similar in both study groups (p > 0.05), with fewer than three patients reporting these symptoms in each group, and none reported after the first hour postoperatively. Thus, the efficacy of 4 mg dexamethasone in preventing PONV was comparable to the 8 mg dose. Our findings align with previous studies [8,9,11]. No other adverse effects were noted in either group.

There was a significant increase in blood sugar levels over time in both groups, with statistically significant differences between the groups at six, 12, 18, and 24 hours. Blood sugar levels rose more in the 8 mg dose group than in the 4 mg group (p < 0.05). These findings are consistent with a study by Purushothaman et al., who concluded that dexamethasone raised blood sugar levels compared to placebo, with a greater magnitude of rise seen with the 8 mg dose than with 4 mg (p < 0.001). They found that dexamethasone 8 mg caused a greater hyperglycemic response in both nondiabetic and diabetic patients after eight hours of administration [15]. Rahimzadeh et al. also observed that the maximum rise in blood sugar occurred in patients receiving the 8 mg dose compared to the 4 mg group, with both 4 mg and 8 mg dexamethasone increasing blood sugar levels compared to the control group at six and 24 hours post-surgery [11].

Limitations

There are several limitations to our study. First, we were unable to assess the prolonged effects of the two different doses on pain and blood sugar levels, as the follow-up period was limited to 24 hours. Additionally, we did not include a placebo control group, which would have allowed us to better evaluate the impact of surgical stress on blood sugar levels. Furthermore, the potential influence of variations in surgical skills between surgeons on VAS scores, as well as the effect of parity (multipara vs. nullipara) on VAS scores, was not considered. Therefore, the results should be interpreted with these limitations in mind.

## Conclusions

The analgesic efficacy of 4 mg IV dexamethasone was comparable to that of 8 mg, as demonstrated by similar pain levels in the postoperative period following LSCS under SA. Additionally, 4 mg IV dexamethasone showed equivalent effectiveness to the 8 mg dose in preventing PONV. However, there was a notable rise in blood sugar levels over time in both groups, with statistically significant differences observed at six, 12, 18, and 24 hours. A greater increase in blood sugar was seen with the 8 mg dose, while the 4 mg dose resulted in a less pronounced elevation (p < 0.05). No clinically or statistically significant differences were found between the two doses regarding the duration of sensory (p = 0.233) or motor block (p = 0.220).

We recommend 4 mg IV dexamethasone as a preferable alternative to the 8 mg dose for patients undergoing LSCS under SA, as it effectively alleviates postoperative pain and reduces the incidence of PONV. Further studies should explore the optimal dosing of dexamethasone across various doses.
